# Supplementary material for: Antimicrobial Activity of the Green Tea Polyphenol (−)-Epigallocatechin-3-Gallate (EGCG) against Clinical Isolates of Multidrug-Resistant Vibrio cholerae
Source: Antibiotics (Basel). 2022 Apr 13;11(4):518. doi: 10.3390/antibiotics11040518 (PMC9028445; doi:10.3390/antibiotics11040518)
Supplement: Supplementary file 1 [file antibiotics-11-00518-s001.zip › antibiotics-1656699-supplementary.pdf]

antibiotics

Supplementary Materials

Antimicrobial Activity of the Green Tea Polyphenol (−)-Epigallocatechin-3-Gallate (EGCG) against Clinical Isolates of Multidrug-Resistant *Vibrio cholerae*

Achiraya Siriphap [1,†], Anong Kiddee [1,†], Acharaporn Duangjai [1,2], Atchariya Yosboonruang [1], Grissana Pook-In [1], Surasak Saokaew [3,4,5], Orasa Sutheinkul [6] and Anchalee Rawangkan [1,2,5,*]

1 School of Medical Sciences, University of Phayao, Phayao 56000, Thailand; achiraya.si@up.ac.th (A.S.); anong.ki@up.ac.th (A.K.); achara.phso@gmail.com (A.D.); f.atchariya@hotmail.com (A.Y.); krissy_seven@hotmail.com (G.P.-I.)
2 Unit of Excellence in Research and Product Development of Coffee, Division of Physiology, School of Medical Sciences, University of Phayao, Phayao 56000, Thailand
3 Division of Social and Administrative Pharmacy, Department of Pharmaceutical Care, School of Pharmaceutical Sciences, University of Phayao, Phayao 56000, Thailand; saokaew@gmail.com
4 Center of Health Outcomes Research and Therapeutic Safety (Cohorts), School of Pharmaceutical Sciences, University of Phayao, Phayao 56000, Thailand
5 Unit of Excellence on Clinical Outcomes Research and Integration (UNICORN), School of Pharmaceutical Sciences, University of Phayao, Phayao 56000, Thailand
6 Faculty of Public Health, Mahidol University, Bangkok 10400, Thailand; orasa.sut@mahidol.ac.th
* Correspondence: ewmedsci@gmail.com; Tel.: +66-5446-6666 (ext. 3824) or +66-86-926-2448
† These authors contributed equally to this work

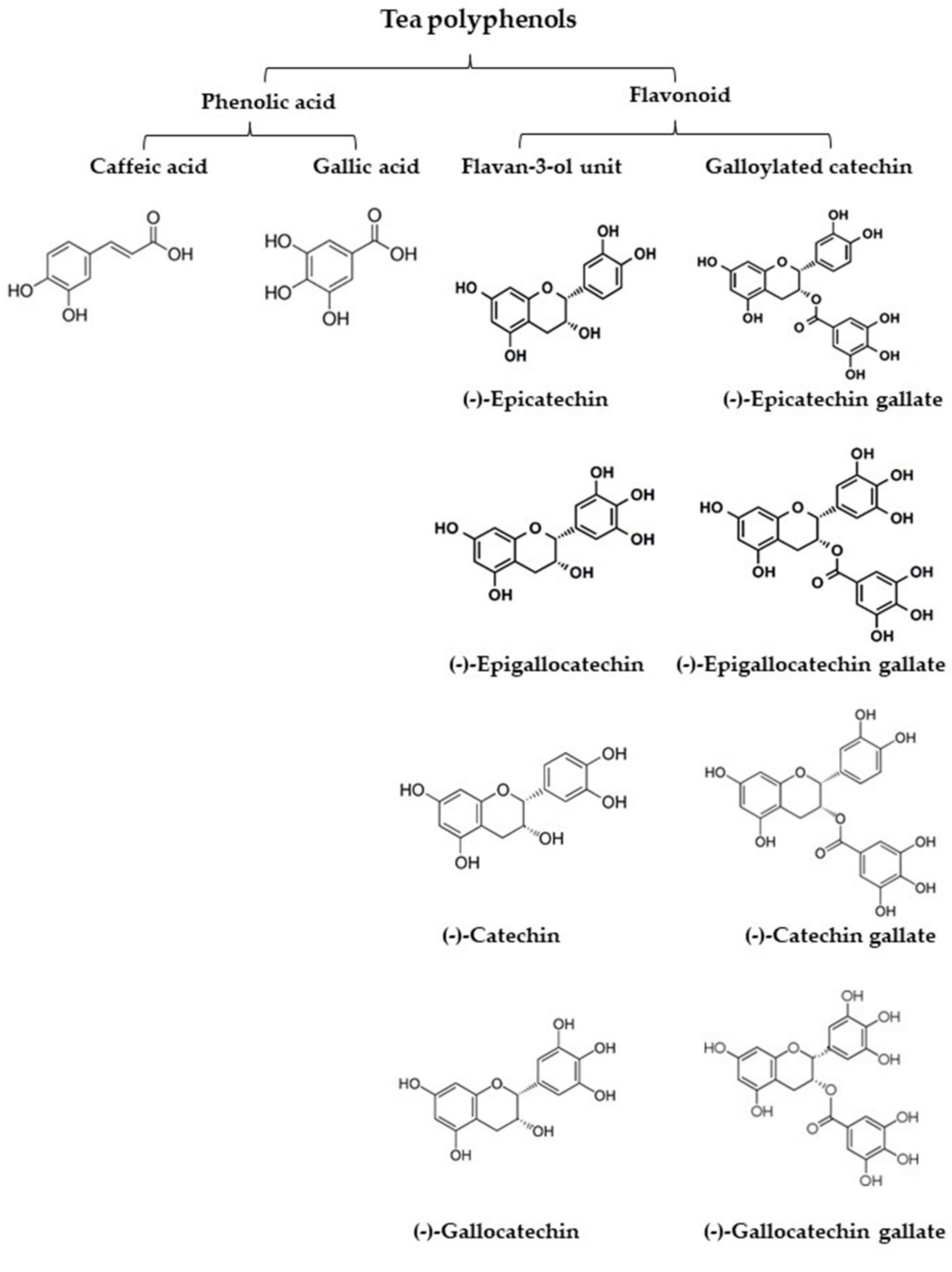

Figure S1. Chemical structures and classifications of tea polyphenols. (Rawangkan A. Mechanistic basis of lung cancer prevention with green tea catechins focused on anti-tumor immunity. Thesis, 2018, doi.org/10.24561/00018648).

Table S1. The susceptibility of a total of 45 *V. cholerae* clinical strains to EGCG (μg/mL).

No. of strains (%)					
**MIC**			**MBC**		
**62.5**	**125**	**250**	**125**	**250**	**500**
15 (33.33)	29 (64.44)	1 (2.22)	15 (33.33)	29 (64.44)	1 (2.22)

Abbreviations: EGCG, (−)-epigallocatechin gallate; MIC, minimum inhibitory concentration; MBC, minimum bactericidal concentration.

Table S2. MIC and MBC of tetracycline for a total of 45 clinical strains of drug-resistant *V. cholerae* strains.

Strain	Serogroup/Serotype/Serovar	MIC (μg/mL)	MBC (μg/mL)
N16961	*V. cholerae* O1 El Tor Inaba	3.91	7.81
P33	*V. cholerae* O1 El Tor Ogawa	15.63	31.25
P34	*V. cholerae* O1 El Tor Inaba	0.48	0.97
P35	*V. cholerae* O1 El Tor Inaba	0.97	1.95
P36	*V. cholerae* O1 El Tor Ogawa	3.91	7.81
P38	*V. cholerae* O1 El Tor Inaba	0.48	0.97
P39	*V. cholerae* O1 El Tor Inaba	7.81	15.63
P41	*V. cholerae* O1 El Tor Ogawa	7.81	31.25
P42	*V. cholerae* O1 El Tor Inaba	0.97	1.95
P43	*V. cholerae* O1 El Tor Inaba	0.48	0.97
P44	*V. cholerae* O1 El Tor Inaba	1.95	3.91
P45	*V. cholerae* O1 El Tor Ogawa	15.63	31.25
P46	*V. cholerae* O1 El Tor Inaba	0.48	1.95
P47	*V. cholerae* O1 El Tor Ogawa	0.48	0.97
P48	*V. cholerae* O1 El Tor Ogawa	62.5	125.0
22115	*V. cholerae* O1 El Tor Inaba	0.48	0.97
22116	*V. cholerae* O1 El Tor Inaba	0.48	0.97
22118	*V. cholerae* O1 El Tor Inaba	0.97	1.95
22125	*V. cholerae* O1 El Tor Ogawa	0.48	0.97
22126	*V. cholerae* O1 El Tor Ogawa	15.63	31.25
22127	*V. cholerae* O1 El Tor Ogawa	0.48	0.97
22135	*V. cholerae* O139	1.95	3.91
22136	*V. cholerae* O139	0.78	1.56
22137	*V. cholerae* O139	1.95	3.91
22138	*V. cholerae* O139	7.81	15.63
22144	*V. cholerae* non-O1, non-O139	0.97	1.95
4053022001	*V. cholerae* O1 El Tor Ogawa	1.95	3.91
4053023816	*V. cholerae* O1 El Tor Inaba	0.48	0.97
4053023817	*V. cholerae* O1 El Tor Ogawa	0.48	0.97
4053023818	*V. cholerae* O1 El Tor Inaba	1.95	3.91
4053023822	*V. cholerae* O1 El Tor Ogawa	15.63	31.25
4053023823	*V. cholerae* O1 El Tor Inaba	3.91	15.63
4053023826	*V. cholerae* O1 El Tor Inaba	0.97	1.95
4053023828	*V. cholerae* O1 El Tor Inaba	15.6	31.25
4053023829	*V. cholerae* O1 El Tor Inaba	1.95	3.91
4053023830	*V. cholerae* O1 El Tor Ogawa	0.97	1.95
4053024283	*V. cholerae* O1 El Tor Inaba	0.97	1.95
4053024290	*V. cholerae* O1 El Tor, Inaba	3.91	7.81
4053024292	*V. cholerae* O1 El Tor Inaba	0.48	0.97
4053024293	*V. cholerae* O1 El Tor Inaba	3.91	7.81
4053024294	*V. cholerae* O1 El Tor Inaba	0.48	0.97
4053024295	*V. cholerae* O1 El Tor Inaba	0.97	1.95
4053024296	*V. cholerae* O1 El Tor Inaba	0.48	1.95
4053024297	*V. cholerae* O1 El Tor Inaba	0.97	3.95

4053024299	*V. cholerae* O1 El Tor Inaba	1.95	3.91

Abbreviations: MIC, minimum inhibitory concentration; MBC, minimum bactericidal concentration. Tetracycline was a two-fold serial dilution from a concentration of 1.0 mg/mL to 0.97 μg/mL in Mueller Hinton Broth (MHB). MHB media solution was used in place of tetracycline as a negative control.
